# Intubation in propofol-treated status epilepticus: a cohort study

**DOI:** 10.3389/fmed.2025.1533753

**Published:** 2025-04-07

**Authors:** Maya Mikutra-Cencora, Jeanne Teitelbaum

**Affiliations:** ^1^Faculty of Medicine, Université de Montréal, Montreal, QC, Canada; ^2^Montreal Neurological Institute, McGill University, Montreal, QC, Canada

**Keywords:** status epilepticus, seizure, endotracheal intubation, propofol, cohort study

## Abstract

**Introduction:**

The management of status epilepticus (SE) often includes endotracheal intubation with mechanical ventilation to address respiratory depression, especially in patients treated with third-line anesthetic agents such as propofol. At our center we use sub-anesthetic propofol as a first line anti-epileptic for SE without intubation. We aimed to assess the performance of our treatment algorithm and to determine whether intubation in these patients improves outcomes.

**Methods:**

All adult patients with SE treated with propofol at a tertiary neuro-intensive care unit from 2015 to 2022 were identified through medical records. Survival without new neurological deficits at discharge was the primary outcome; secondary outcomes were the development of common complications. Descriptive statistics were used to assess general outcomes, and multi-variable logistic regressions were performed to compare outcomes between patients who were intubated while on propofol and those who were not, as well as to compare outcomes according to number of days kept intubated after cessation of propofol.

**Results:**

We identified 162 SE patients treated with low-dose propofol, of which 44 (17%) were not intubated and 118 (83%) were. Our cohort’s survival rate was 85%, and survival without new deficits was 42%. Intubation was not associated with improved survival without new neurological deficits (OR = 1.34, 95% CI 0.372–4.831, *p* = 0.655) or reduction in complications. Additionally, in patients intubated for management of SE, the number of days kept intubated following cessation of propofol was associated with a decrease in survival without new neurological deficits (OR = 0.016, 95% CI 0.000–0.854, *p* = 0.042).

**Conclusion:**

This study offers encouraging evidence that early sub-anesthetic propofol is a safe and efficient alternative to existing treatment approaches in selected patients. These patients do not require intubation: intubation fails to significantly improve outcomes, and prolonging intubation past cessation of propofol worsens outcomes. These data raise doubts as to the benefits of endotracheal intubation in SE and stress the need to limit the duration of this invasive measure.

## 1 Introduction

Status epilepticus (SE) is one of the most common neurological emergencies and is associated with high morbidity and mortality, as high as 40% in refractory cases (1).

The definition of SE has changed significantly over time and remains contested (2), as it may include patients who may not be as severely affected. Time frames and risk of permanent damage vary according to semiology, and is different for absence status, focal motor status or non-convulsive status. Treatment approaches differ between the different types of SE.

The present operational definition is based on the 2015 proposal by the International League Against Epilepsy Task Force (3). Convulsive (generalized tonic–clonic) SE (CSE) is defined as constant convulsive activity of more than 5 min or the onset of recurrent (3 or more) brief seizures without return to consciousness between events. Convulsive seizures lasting 5 or more minutes have a very low chance of spontaneous cessation, and if CSE lasts more than 30 min there is compelling evidence of long-term sequelae and increased mortality (3).

Therefore, treatment should be prompt, adequate and evidence-based, aiming at clinical and electroencephalographic cessation as quickly and safely as possible. Management of SE must include three aspects: stop seizures, stabilize patients to avoid secondary lesions and treat underlying causes.

There are many treatment protocols and guidelines for the treatment of convulsive SE, covering the initial, established and refractory stages (4–6). All agree that the longer the duration of the seizures, the more refractory to treatment they will be.

All agree at present that the immediate treatment is the administration of a benzodiazepine, usually lorazepam (4–6). Basic critical care and emergency principles of therapy such as supporting respiration, maintaining blood pressure, gaining intravenous access and treating the underlying cause have achieved widespread acceptance.

The management of benzodiazepine-resistant SE has been evolving, and the approaches to the pharmacologic treatment of SE can vary from one guideline to another. In a systematic review by Jain et al. (7) as well as a previous one by Brigo et al. (8), phenobarbital had the highest probability of being effective, followed by high dose levetiracetam and high dose valproate, and all were significantly superior to phenytoin. Because of tolerability and drug interaction considerations, levetiracetam became the drug of choice for SE resistant to first line benzodiazepine for control of seizures within 30 to 60 min.

In many if not most protocols, propofol and other anesthetic agents are reserved for refractory SE, when first- and second-line therapies have been administered and failed. Intubation and protection of the airway are considered necessary prior to propofol infusion as the suggested doses are high and cause deep sedation (4). By the time one reaches this stage, 40 to 60 min will have elapsed, and there may be permanent CNS damage.

**Figure 1:**
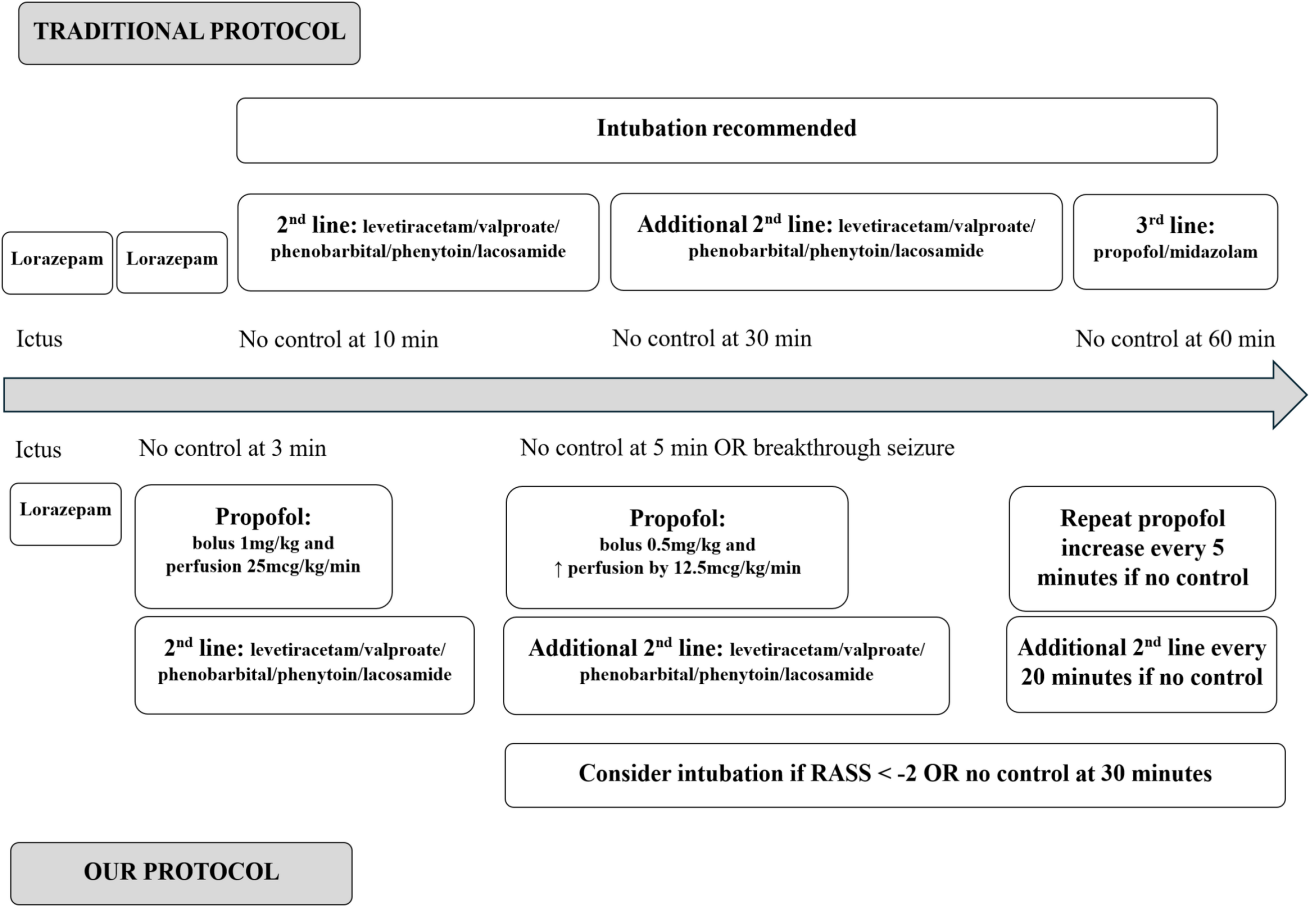
Comparison of traditional management of SE [adapted from Lam et al. (20)] and our treatment protocol.

Propofol has thus mostly been relegated to third-line therapy where it works well. There is evidence however, albeit non-randomized controlled, that non-anesthetic doses of propofol can also control seizures well (9, 10). Therefore, based on the fact that 10% to 30% of patients will not respond to first and second line therapy (11), that continuing seizures will cause permanent damage after 30 min (3), and that propofol can control seizures well, at our institution we use non-anesthetic propofol immediately after benzodiazepine administration to control seizures while administering a second-line agent (levetiracetam, valproate, other) (see Figure 1 for details). Patients are not intubated in this scenario unless ongoing seizures or inadequate airway protection (Richmond Agitation Sedation Scale below −2) require it. For patients received as transfers from other institutions who arrive intubated and on high-dose propofol, we decrease this to low-dose propofol and extubate if there is no seizure recurrence, vital signs are stable and the patient can protect their airway. Agitation after decreasing the propofol level is not a reason to prolong sedation or intubation as long as the patient is able to protect their airway. Intubated patients on high dose propofol with continuing status (electrical or clinical) do not have their propofol dosage decreased and are not extubated.

This protocol differs from traditional guidelines in its use of propofol and its avoidance of intubation. Though intubation is an invasive measure that carries well-known risks (12, 13), and literature has begun to call into question its use in SE (14), even suggesting that it may worsen outcomes (15), many of the SE treatment guidelines cited above encourage endotracheal intubation to protect the airway and assure proper oxygenation. Propofol also has theoretical risks of hypotension, aspiration pneumonia, respiratory insufficiency and propofol infusion syndrome. For these reasons, we set out to examine a cohort of SE patients managed with our treatment algorithm, using sub-anesthetic propofol while avoiding intubation. Our objectives were to determine (1) whether the use of low-dose propofol in our SE treatment algorithm is safe and effective, (2) whether intubation in low-dose propofol-treated SE patients improves outcomes compared to patients treated without intubation, and (3) whether early extubation in low-dose propofol-treated patients who are intubated at admission improves or worsens outcomes.

## 2 Methods

### 2.1 Study population and ethics approval

All patients aged 18 years or older, with SE, treated with propofol, and admitted to the Neuro-Intensive Care Unit of the Montreal Neurological Institute (Montreal, Canada) between January 1, 2015, and June 18, 2022, were included in this study. SE was confirmed in all patients clinically or electrographically according to the definition of SE outlined in the 2015 proposal by the International League Against Epilepsy Task Force (3).

This study received ethics approval from the McGill University Health Centre Research Ethics Board (project number 2024-9974) on January 8, 2024 under the study title “Intubation in propofol-treated status epilepticus: a cohort study.” Procedures were followed in accordance with the ethical standards of the responsible committee on human experimentation and with the Helsinki Declaration of 1975.

### 2.2 Data collection

All data were gathered retrospectively from medical records by one investigator (MMC), with an additional review of data flagged as uncertainties by a second investigator (JT). Data were collected for the following variables: patient demographics (sex, age), past medical history [comorbidities were assessed using the Charlson Comorbidity Index (16)], admission type (local admission or transfer), characteristics of SE (type, suspected cause, EEG characterization when available), endotracheal intubation & mechanical ventilation (intubation status, reason for intubation, duration of intubation, duration of intubation following cessation of propofol administration), pharmacological treatment of SE [anti-epileptic drugs (AEDs) administered, duration of administration of propofol, other drugs administered for SE], outcomes (survival, survival without new neurological deficits at discharge, in-hospital adverse events).

### 2.3 Statistical analysis

Descriptive statistics were used to summarize demographic and clinical characteristics of all patients. We also described outcomes of our entire cohort in order to evaluate the performance of our treatment protocol.

Next, for our primary analysis, patients were separated into two groups according to intubation status (intubated during hospital stay while on low dose propofol, or not intubated during hospital stay). Uni- and multi-variable logistic regressions were performed to compare outcomes of survival, survival without new neurological deficits, and most common in-hospital adverse events (as less common adverse events did not provide enough statistical power for analysis). Control variables were chosen according to clinical relevance and differences in summary descriptive statistics between groups.

In order to assess the risks and benefits of early extubation, additional analyses were performed within the group of patents intubated while receiving low-dose propofol via uni- and multi-variable logistic regressions. Our secondary analysis compared the afore-mentioned outcomes in patients extubated while still receiving low-dose propofol and patients extubated following the cessation of low-dose propofol administration. A tertiary analysis compared the same outcomes according to number of days kept intubated after cessation of propofol administration (excluding patients re-intubated for new reasons unrelated to SE). Control variables for these analyses were chosen as described above.

## 3 Results

A total of 162 SE patients treated with propofol were included. Summary characteristics are shown in Table 1. 79% of patients (*n* = 118) were intubated and mechanically ventilated.

**Table 1:** Summary of patient characteristics.

		Never intubated	Intubated		
			Total	Extubated on propofol	Not extubated on propofol
*n*		44	118	33	85
Sex (female)		27 (61%)	57 (48%)	15 (46%)	42 (49%)
Age (range)		54 (18–87)	52 (18–92)	51 (20–84)	52 (18–92)
Charlson comorbidity index (range)		3.61 (0–12)	2.86 (0–10)	2.61 (0–7)	2.96 (0–10)
Chronic, static, or progressive encephalopathy		9 (21%)	11 (9%)	3 (9%)	8 (9%)
History of epilepsy/seizures		27 (61%)	55 (47%)	19 (58%)	37 (44%)
Controlled epilepsy? (i.e., <1/month or mention of “refractory,” “drug-resistant”)		8 (18%)	22 (19%)	8 (24%)	14 (17%)
Local admission (vs. transfer)		29 (66%)	29 (25%)	4 (12%)	25 (30%)
SE type	Convulsive (generalized)	3 (7%)	54 (46%)	15 (46%)	39 (46%)
	Convulsive (focal)	24 (55%)	21 (18%)	4 (12%)	17 (20%)
	Convulsive (focal and generalized)	6 (14%)	18 (15%)	3 (9%)	15 (18%)
	Convulsive (not described)	1 (2%)	5 (4%)	3 (9%)	2 (2%)
	Convulsive (generalized) and non-convulsive	1 (2%)	5 (4%)	3 (9%)	2 (2%)
	Convulsive (focal) and non-convulsive	1 (2%)	7 (6%)	2 (6%)	5 (6%)
	Non-convulsive (focal, impaired awareness)	4 (9%)	6 (5%)	3 (9%)	2 (4%)
	Non-convulsive (generalized, impaired awareness)	4 (9%)	1 (1%)	0 (0%)	1 (1%)
	Not described	0 (0%)	1 (1%)	0 (0%)	1 (1%)
Suspected cause of SE	Adverse drug reaction	1 (2%)	0 (0%)	0 (0%)	0 (0%)
	AED-related	1 (2%)	16 (14%)	6 (18%)	10 (12%)
	Brain hemorrhage	5 (11%)	15 (13%)	3 (9%)	12 (14%)
	Brain ischemia	1 (2%)	2 (2%)	1 (3%)	1 (1%)
	Brain tumor	10 (22%)	24 (20%)	7 (21%)	17 (20%)
	CNS infection	2 (5%)	11 (9%)	2 (6%)	9 (11%)
	Non-infectious encephalitis	0 (0%)	3 (3%)	0 (0%)	3 (4%)
	Psychoactive substance-related	0 (0%)	4 (3%)	1 (3%)	3 (4%)
	Usual epilepsy triggers	6 (14%)	9 (8%)	4 (12%)	5 (6%)
	Other	5 (11%)	11 (9%)	2 (6%)	9 (11%)
	Unclear	13 (30%)	23 (20%)	7 (21%)	16 (19%)
Number of AEDs administered		3.7 (1–9)	3.5 (1–10)	3.18 (1–8)	3.62 (1–10)
Other treatments administered: ketamine		2 (5%)	20 (17%)	5 (15%)	15 (18%)
Reason for intubation	SE-related	N/A	98 (83%)	27 (82%)	71 (84%)
	Other (agitation)	N/A	2 (2%)	1 (3%)	1 (1%)
	Other (neurologic)	N/A	5 (4%)	3 (9%)	2 (2%)
	Other (respiratory)	N/A	11 (9%)	1 (3%)	10 (12%)
	Other (post-op complications)	N/A	1 (1%)	0 (0%)	1 (1%)
	Unclear	N/A	1 (1%)	1 (3%)	0 (0%)

The intubated patients in our study are heterogeneous. If these patients arrive intubated from another institution, at the Montreal Neurological Institute, one group of these patients is immediately extubated after decreasing the propofol to sub-anesthetic levels if they are seizure-free and able to protect their airway, while the other group remains intubated for seizure control or respiratory necessity.

Patients who were never intubated, compared to those who were, were more often female, more often had a history of chronic, static, or progressive encephalopathy, and more often had a history of epilepsy/seizures. Patients who were never intubated were also less often transferred from other centers and less often treated with ketamine as part of their SE management. The type of seizure was more often focal tonic–clonic rather than generalized, and SE was less often AED compliance-related in etiology. Patients who were never intubated had a similar average Charlson Comorbidity Index to those who were intubated.

Within the group of intubated patients, those extubated early while remaining on low-dose propofol (compared to those kept intubated) more often have been transferred from another site, have a history of epilepsy/seizures, and were often intubated for reasons other than control of seizures (level of consciousness, protection of airway for transport, agitation). Patients extubated while on low-dose propofol had a similar average Charlson Comorbidity Index to those who were kept intubated, and had a similar proportion of AED compliance-related SE.

Patients who had non-infectious encephalitis (*n* = 3) were all intubated and were not extubated while on propofol (were kept intubated longer).

The variables for which the above-mentioned differences are noted were included as co-variates in our statistical analyses (see below).

### 3.1 Descriptive assessment: performance of our treatment algorithm

Additional information regarding the administration of our treatment algorithm can be found in Table 2. Prior to the administration of low-dose propofol, the majority of patients received a benzodiazepine, and approximately half received a benzodiazepine followed by additional agents. The mean total number of agents given prior to propofol administration was 2, with a range from 0 to 5.

**Table 2:** Additional characteristics of pharmacological treatment in unintubated patients.

	Never intubated		
	Total (*n* = 42)	Local (*n* = 29)	Transfer (*n* = 13)^*^
Duration of propofol administration (until SE control) (hours:min, mean, range)	3:59 (0:02–25:55)	3:34 (0:02–25:55)	4:43 (0:02–21:00)
AEDs administered before propofol (mean, range)	2.0 (0–5)	1.8 (0–4)	2.5 (0–5)
Benzodiazepine	36 (86%)	25 (86%)	11 (85%)
Additional AED after benzodiazepine	23 (55%)	14 (48%)	8 (62%)
Phenytoin	20 (48%)	12 (42%)	8 (62%)
Levetiracetam	18 (43%)	11 (38%)	7 (54%)
Lacosamide	5 (12%)	1 (3%)	4 (31%)
Carbamazepine	4 (10%)	4 (14%)	0 (0%)
Phenobarbital	4 (10%)	1 (3%)	3 (23%)
Lamotrigine	1 (2%)	0 (0%)	1 (8%)
Valproate	1 (2%)	0 (0%)	1 (8%)
AEDs administered after propofol (mean, range)	0.6 (0–4)	1.0 (0–3)	0.5 (0–2)
Any AED	25 (60%)	20 (69%)	5 (38%)

^*^Missing detailed pharmacological data in chart review for 2 patients.

In the minority of patients that did not receive a benzodiazepine before propofol, this was mainly due to the fact that these patients were refractory epileptics who received their multiple home AEDs prior to arrival.

Total mortality for all patients was 15% (survival 85%, *n* = 138), and survival without new neurological deficits at discharge was 42% (*n* = 68). Fifty-seven (57) percent of patients (*n* = 92) presented at least one adverse event in-hospital, the most common of these being pneumonia, sepsis, and delirium or delirium-like symptoms (agitation, confusion, hallucinations, etc.) (see Table 3).

**Table 3:** Summary of patient outcomes.

Outcomes	Our cohort
Survival	85%
Survival without new deficits	42%
In-hospital adverse events	57%

In addition, we assessed the prevalence of adverse outcomes that have traditionally been associated with the use of propofol without intubation (see Table 4): subsequent urgent intubation for sedation-induced lack of airway protection (as opposed to lower level of consciousness due to resistant SE), refractory hypotension, apneic episodes, and propofol infusion syndrome. These events were very rare in our cohort. For subsequent intubation, none of the three cases were linked to the use of propofol. One patient was initially not intubated while receiving low dose propofol, then intubated 4 days post propofol cessation for a decrease in level of consciousness due to phenobarbital and pneumonia. A second patient was initially intubated, extubated while receiving low dose propofol, then subsequently reintubated for desaturation. This was followed by successful extubation 4 days later while still receiving low dose propofol. A third patient who was initially intubated had a failed first attempt at extubation due to stridor but was then successfully extubated.

**Table 4:** Summary of adverse outcomes traditionally linked to use of propofol without intubation.

Outcomes	Initially not intubated	Arrived intubated, extubated early
	*n* = 44	*n* = 33
Subsequently intubated	1	2
Refractory hypotension	0	4
Apneic episode	0	0
Propofol infusion syndrome	0	0

### 3.2 Primary analysis: according to intubation status

Based on clinical relevance and differences in summary statistics between intubated and non-intubated groups (see Table 1), multi-variable logistic regression included the following potential confounders as co-variables: sex, age, Charlson Comorbidity Index, pre-existing encephalopathy, previous history of epilepsy (controlled or not), local admission or transfer, type of SE, cause of SE, number of AEDs administered, and administration of ketamine. As shown in Table 5, intubation was not associated with improved survival (OR = 1.077, 95% CI 0.243–4.775, *p* = 0.922) or survival without new neurological deficits (OR = 1.340, 95% CI 0.372–4.831, *p* = 0.655). It was also not associated with a reduction in the most common adverse events: pneumonia (OR = 0.940, 95% CI 0.340–2.601, *p* = 0.905), sepsis (OR = 1.658, 95% CI 0.257–10.694, *p* = 0.595), and delirium/agitation (OR = 1.311, 95% CI 0.260–6.618, *p* = 0.743).

**Table 5:** Effect of intubation status on outcomes.

	Unadjusted models			Multi-variable adjusted models		
Outcomes	OR	95% confidence interval	*p*-value	OR	95% confidence interval	*p*-value
Survival without new neurological deficits	1.808	0.871–3.754	0.112	1.34	0.372–4.831	0.655
Survival	0.877	0.324–2.376	0.797	1.077	0.243–4.775	0.922
Pneumonia	1.376	0.612–3.093	0.44	0.94	0.340–2.601	0.905
Sepsis	1.692	0.458–6.248	0.43	1.658	0.257–10.694	0.595
Delirium/agitation	2.959	0.836–10.469	0.092	1.311	0.260–6.618	0.743

### 3.3 Secondary analysis: according to extubation before or after propofol cessation

Multi-variable logistic regression included the same co-variables as for our primary analysis, as well as an additional co-variable of reason for intubation. As shown in Table 6, patients extubated during continuing low dose propofol administration did not present decreased survival (OR = 0.999, 95% CI 0.220–4.527, *p* = 0.999) or decreased survival without new neurological deficits (OR = 66.449, 95% CI 0.613–7198.619, *p* = 0.079), as may have been feared. Extubation during continuing low dose propofol did not however improve these outcomes. With regard to outcomes of adverse events, pneumonia and delirium/agitation were sufficiently frequent to allow statistical analysis. Extubation during low-dose propofol was not associated with any increase in likelihood of pneumonia (OR = 0.452, 95% CI 0.117–1.747, *p* = 0.250). However, patients extubated during continuing low-dose propofol administration presented an increase in delirium/agitation (OR = 8.875, 95% CI 1.719–45.810, *p* = 0.009).

**Table 6:** Effect of extubation before propofol cessation on outcomes.

	Unadjusted models			Multi-variable adjusted models		
Outcomes	OR	95% confidence interval	*p*-value	OR	95% confidence interval	*p*-value
Survival without new deficits	1.939	0.859–4.377	0.111	0.999	0.220–4.527	0.999
Survival	3.594	0.778–16.596	0.101	66.449	0.613–7198.619	0.079
Pneumonia	0.578	0.223–1.497	0.259	0.452	0.117–1.747	0.25
Delirium/agitation	3.75	1.408–9.986	0.008	8.875	1.719–45.810	0.009

### 3.4 Tertiary analysis: according to number of days intubated following propofol cessation

Multi-variable logistic regression included the same co-variables as for our secondary analysis, as well as additional co-variables for presence of pneumonia, sepsis, and delirium/agitation, as these adverse events are common causes for prolonged intubation. As shown in Table 7, the number of days kept intubated following cessation of low dose propofol was associated with decreased survival without new neurological deficits (OR = 0.014, 95% CI 0.000–0.803, *p* = 0.039) (analysis for other outcomes was not done due to insufficient statistical power).

**Table 7:** Effect of duration of intubation past propofol cessation on outcomes.

	Unadjusted models			Multi-variable adjusted models		
Outcomes	OR	95% confidence interval	*p*-value	OR	95% confidence interval	*p*-value
Survival without new deficits	0.269	0.111–0.647	0.003	0.016	0.000–0.854	0.042

## 4 Discussion

In this retrospective study, we examined a cohort of SE patients treated with sub-anesthetic doses of propofol. Our goals were to describe the performance of this treatment algorithm and determine the usefulness of intubation in these patients.

Our study confirms existing data showing that SE is a high-mortality, high-morbidity pathology, with generally poor outcomes. This has previously been shown by multiple studies examining outcomes of patients managed with current treatment guidelines. To better evaluate the value of our treatment algorithm, using administration of sub-anesthetic propofol, we compared our cohort’s general outcomes to existing literature on current algorithms (17). Both perform similarly in terms of survival outcomes (with survival rates of 85% for our cohort and 83% for cohorts managed with current guidelines). This is encouraging preliminary evidence to support the use of early low-dose propofol as an alternative to current treatment algorithms in SE in selected patients. Further analytical studies are required to confirm this.

It must however be noted, that our treatment protocol is restricted to a pool of patients whose clinical evolution allows for avoidance of intubation and whose outcome is likely to be better than that of the entire group of SE. The differences between our series of patients and other epidemiological assessments of SE cohorts highlight the importance of patient triage in the use of our treatment algorithm in emergency departments, to optimize its safety.

It is also important, within our treatment algorithm, to identify failures of low-dose propofol and indications for escalation of treatment. Indeed, within the sub-group of patients who were never intubated (analyzed as a clearer representation of our protocol’s performance), our data shows that the duration from initiation of propofol to SE control varies considerably, from minutes to over 24 h, and the majority of patients required an additional agent following propofol administration. This highlights the need for these additional treatment options and clear criteria for when they should be administered following propofol. In our current treatment algorithm (see Figure 1), these additional agents are administered every 20 min if SE control is not achieved after initiation of propofol.

In addition to evaluating the performance of sub-anesthetic propofol in SE, we also examined the place of intubation within this treatment algorithm. Our descriptive data show that intubation in SE patients is very common in our setting. It is difficult to determine whether this is a generalized phenomenon, as intubation rates in existing literature vary (15, 18), but the widespread use of intubation in SE management likely reflects the fact that current guidelines often recommend this measure. Our descriptive data also supports this influence of current guidelines, as we find that patients who are not intubated are more likely to have focal than generalized SE—guidelines do not recommend intubation in these patients because of lesser severity of disease. Our descriptive data also provide insight into another rationale that may drive decisions to intubate in SE. We found that patients who are not intubated are more likely have a history of seizures or epilepsy, or of chronic encephalopathy. This may be because these types of patients more often have had SE in the past or have refractory epilepsy, making providers less alarmed by the current episode of SE, and thus less likely to intubate. These data suggest that the decision to intubate is not only guided by recognized treatment algorithms but is also influenced by individual physicians’ levels of comfort with the specific characteristics of each SE case. This conclusion is also supported by previous literature that finds that decisions on intubation in SE greatly vary depending on treating teams (19).

Next, data from our study provide several conclusions on the necessity of intubation in SE patients treated with low-dose propofol. We found that intubation does not improve outcomes in these patients. Thus, SE can be safely treated with sub-anesthetic propofol without intubation and mechanical ventilation. For patients who are already intubated at admission, extubation can be safely performed while continuing sub-anesthetic propofol administration. Clinicians should however be aware that these extubated patients are at increased risk of developing delirium or agitation. These do not require re-intubation (which, as mentioned, does not improve outcomes). In fact, intubation should not be prolonged past the cessation of propofol (unless a valid indication persists), as this worsens outcomes. This conclusion is particularly important, as previous literature has shown extubation is often delayed without valid indications in SE patients (15).

In summary, this study thus shows the potential of using early sub-anesthetic propofol, without intubation (or with early extubation), as a safe and effective agent in SE management.

This study does have certain limitations. It is a retrospective, single-center study with a relatively small sample size. Also worth noting is that the majority of patients in our cohort are transfers from other institutions: thus, initial treatment is not according to our protocol. This however also makes our data more representative of how our treatment protocol would perform if put in place in other tertiary institutions. Finally, a bias of confounding by indication exists in our analysis (i.e., patients are intubated because their SE is more severe, and so intubated patients will have worse outcomes). Though this bias cannot be completely overcome, we addressed it by including a wide range of co-variables reflecting severity of disease in statistical analyses.

## 5 Conclusion

This study offers encouraging evidence that early sub-anesthetic propofol is a safe and efficient alternative to existing treatment approaches in selected patients, with the potential to be used as a first-line agent in SE. Our data also raises doubts as to the necessity and benefits of endotracheal intubation in these patients and stresses the need to limit the duration of this invasive measure frequently included in the management of SE. Our data show that in SE treated with low-dose propofol, intubation fails to significantly improve outcomes, and prolonging this measure after the cessation of propofol worsens outcomes. These data provide an alternate guideline to clinicians, in first-line as well as intensive care settings, with regards to a pathology for which current management algorithms have failed to produce an acceptable mortality and morbidity profile.

## Data Availability

The raw data supporting the conclusions of this article will be made available by the authors, without undue reservation.
